# Examination of the proportion and characteristics of cognitive function changes during hospitalization in patients with cardiovascular diseases

**DOI:** 10.1371/journal.pone.0309306

**Published:** 2024-08-22

**Authors:** Takahiro Shimoda, Shinzi Suzuki, Daisuke Mizukoshi, Wada Saori, Erina Yokoshima, Tomoko Terai

**Affiliations:** 1 Department of Preventive Gerontology, Center for Gerontology and Social Science, National Center for Geriatrics and Gerontology, Aichi, Japan; 2 Department of Rehabilitation, Tokyo Metropolitan Police Hospital, Tokyo, Japan; 3 Department of Cardiology, Tokyo Metropolitan Police Hospital, Tokyo, Japan; Tokyo Metropolitan Institute of Geriatrics and Gerontology, JAPAN

## Abstract

**Objective:**

Cognitive function decline is influenced by cardiovascular diseases and associated risk factors. However, changes in the cognitive function of patients with cardiovascular diseases during hospitalization and the factors influencing these changes remain unclear. This study elucidated the proportion and characteristics of changes in cognitive function during hospitalization in patients with cardiovascular diseases.

**Methods:**

We conducted cognitive function assessments at admission and discharge for patients with cardiovascular diseases. Using the Mini-Mental State Examination (MMSE) and the Japanese version of the Montreal Cognitive Assessment (MoCA-J), we categorized the patients into cognitive impairment, mild cognitive impairment (MCI), and non-cognitive impairment. Changes in MMSE or MoCA-J scores of ≥2 points at discharge were classified as improvement or decline, and all others as maintenance.

**Results:**

The cognitive impairment, MCI, and non-cognitive impairment categories comprised 215 (41.3%), 224 (40.2%), and 103 (18.5%) patients, respectively. The results of the cognitive function assessment at the time of discharge classified 90 patients (35.9%) into the maintenance group, 117 (46.6%) into the improvement group, and 44 (17.5%) into the decline group based on changes during hospitalization. There was a statistically significant difference among the three groups only in cognitive function at admission (P = 0.026). In multivariate analysis, those with MCI or cognitive impairment at admission and younger patients were associated with improved cognitive function during hospitalization. No factors were extracted that showed statistically significant associations with cognitive decline.

**Conclusion:**

Approximately half of the patients with cardiovascular disease experienced improvements in cognitive function during hospitalization, while approximately 20% showed a decline in cognitive function during the same period. These findings demonstrate the importance of assessing cognitive changes in hospitalized patients with cardiovascular disease. Future studies are needed to identify factors associated with changes in cognitive function.

## Introduction

In Japan, the aging population of individuals with cardiovascular diseases has increased [1], and the prevalence of cognitive impairment among these patients has become a significant concern [2]. Patients with cardiovascular disease who experience cognitive dysfunction have a poor life prognosis [3], increased risk of rehospitalization [4], and decreased activities of daily living (ADL) functionality [5]. Therefore, the assessment and intervention of cognitive function in patients with cardiovascular disease are recommended in rehabilitation guidelines for cardiovascular diseases [6].

Cardiovascular diseases and associated risk factors have been reported as contributors to age-related cognitive decline [7,8]. Factors such as bed rest during hospitalization and environmental changes pose risks for cognitive impairment [9,10]. The cognitive function of patients with cardiovascular disease may decline during hospitalization. However, treatments for cardiovascular disease often result in increased cardiac output and cerebral blood flow [11,12]. Previous studies have indicated increased cerebral blood flow is associated with improved cognitive function [13–15]. Therefore, treatment may improve cognitive function in patients with cardiovascular disease.

However, evidence regarding cognitive function changes in patients with cardiovascular disease during hospitalization remains insufficient. Research reporting changes in cognitive function during hospitalization for patients with cardiovascular disease is scarce, and the factors influencing these changes still need to be clarified. Consequently, this study aimed to elucidate the percentage of patients with cardiovascular disease experiencing changes in cognitive function during hospitalization and identify the characteristics associated with such changes.

## Materials and methods

We accessed medical records to conduct this retrospective study on August 28, 2022. The study population consisted of patients admitted to the Cardiology Department of our hospital from September 2019 to March 2022 who underwent cardiac rehabilitation interventions and for whom cognitive function assessment at the time of admission was feasible. Individuals who declined cognitive function assessment, those with severe visual impairment or hearing difficulties hindering assessment, those discharged within one week of the initial cognitive function evaluation, and those with dementia were excluded.

On admission, cognitive function, physical function, and clinical background factors were assessed, with a follow-up cognitive function reassessment at discharge. Cognitive function was evaluated using the Mini-Mental State Examination (MMSE) [16] and the Japanese version of the Montreal Cognitive Assessment (MoCA-J) [17]. Initially, the MMSE was conducted upon admission, and individuals with scores ≤23 were classified as having cognitive impairment [16,18]. Individuals scoring ≥24 underwent subsequent evaluation with the MoCA-J. MoCA-J scores ≤25 indicated mild cognitive impairment (MCI), and scores ≥26 indicated non-cognitive impairment [17]. Individuals in the cognitive impairment group underwent MMSE reassessment at discharge, whereas those in the MCI and non-cognitive impairment groups underwent MoCA-J reassessment. Improvement was defined as a change of ≥2 points in MMSE or MoCA-J scores from those at admission; individuals showing an increase were categorized as the improvement group, those showing a decrease as the decline group, and the remainder as the maintenance group [19–22]. Occupational therapists performed the cognitive function tests according to the manual. Physical function was assessed using grip strength and the Short Physical Performance Battery (SPPB) [23–25].

Clinical background factors extracted from medical records included diagnosis on admission, age, sex, body mass index (BMI), left ventricle ejection fraction, serum albumin (Alb), hemoglobin, brain natriuretic peptide (BNP), C-reactive protein (CRP), estimated glomerular filtration rate (eGFR), Geriatric Nutritional Risk Index (GNRI), admission blood pressure and heart rate, pre-admission ADL (Katz index), presence of dementia, comorbidities (hypertension, diabetes, dyslipidemia, atrial fibrillation, chronic kidney disease, chronic obstructive pulmonary disease, cerebrovascular disease, angina pectoris, myocardial infarction, heart failure admission, lower limb peripheral arterial disease, and malignant tumors), care level, facility admission, employment status, history of falls, length of hospital stay, and outcomes. The GNRI was calculated as 14.89 × serum Alb value + 41.7 × (admission weight/ideal weight) [26].

Comparisons of patient background factors among the cognitive impairment, MCI, and non-cognitive impairment groups at admission were analyzed using the Kruskal–Wallis or χ^2^ test. Three-group comparisons of cognitive function changes were conducted using the Kruskal–Wallis or χ^2^ test for each group based on cognitive function at admission. A multinomial logistic regression analysis was performed using a change in cognitive function during hospitalization as the outcome. Four models were created with a maximum of five independent variables to account for overfitting. Model 1 used severity of cardiac disease, Model 2 used laboratory data, Model 3 used comorbidities, and Model 4 used pre-admission activity status and physical function at admission as independent variables. Comparisons of background factors between those with and without follow-up cognitive function during hospitalization were analyzed using the Mann–Whitney U or χ^2^ test. Statistical analyses were performed using R and EZR software [27]. R is an open-source free software. The statistical significance level was set at 5%.

This study was approved by the Tokyo Police Hospital Ethics Committee (approval number: 19-A20). Medical records were used, and the Ethics Committee waived the requirement for informed consent. All participants were informed of their inclusion in the study and that they were free to withdraw at any time.

## Results

A total of 828 individuals were admitted during the study period, and 611 underwent MMSE assessments. Among these, 215 individuals scored ≤23 on the MMSE, whereas 396 individuals scored ≥24. Among the 396 individuals with MMSE scores ≥24, 327 underwent MoCA-J assessments. Of the total study population, 215 individuals (41.3%) were classified as having cognitive impairment, 224 (40.2%) as having MCI, and 103 (18.5%) as having non-cognitive impairment. Background factors among patients in the three groups are presented in Table 1. Age, sex, BMI, Alb, BNP, CRP, eGFR, GNRI, heart rate, pre-hospitalization Katz index, diabetes, dyslipidemia, cerebrovascular disease, care levels of Japanese public long-term care insurance, nursing home resident, employment status, length of hospital stay, outcomes, grip strength, and SPPB were significantly different among the three groups.

**Table 1:** Patient characteristics stratified by cognitive function at admission.

	Non-cognitive impairment (N = 103)	MCI (N = 224)	Cognitive impairment (N = 215)	P value
Cause of hospitalization (%)				<0.001
Heart failure	71 (68.9)	184 (82.1)	187 (87.0)	
Coronary artery disease	32 (31.1)	35 (15.6)	24 (11.2)	
Other	0 (0.0)	5 (2.2)	4 (1.9)	
Age, years	71 [63–81]	84.00 [76–87]	87 [83–92]	<0.001
Women (%)	30 (29.1)	93 (41.5)	141 (65.6)	<0.001
BMI, kg/m^2^	24.1 [21.3–26.4]	22.9 [20.0–25.5]	21.0 [18.8–24.1]	<0.001
LVEF, %	53.6 [33.7–64.5]	53.2 [34.9–63.8]	54.3 [40.5–65.1]	0.586
Alb, g/dL	3.7 [3.4–4.0]	3.6 [3.3–3.9]	3.4 [3.1–3.7]	<0.001
Hb, g/dL	12.8 [11.4–14.7]	12.0 [10.2–14.0]	11.1 [9.7–12.3]	<0.001
BNP, pg/mL	346.1 [111.9–607.3]	472.1 [221.9–822.6]	478.7 [237.8–969.1]	0.001
CRP, mg/dL	0.4 [0.1–1.6]	0.6 [0.1–2.9]	0.8 [0.2–3.4]	0.012
eGFR, mL/min/1.73 m^2^	51.3 [36.8–66.4]	42.0 [27.4–57.1]	37.0 [25.0–48.9]	<0.001
GNRI, score	99.9 [93.2–109.7]	97.1 [89.5–103.9]	89.8 [84.7–97.7]	<0.001
SBP, mmHg	133 [114–155]	139 [118–161]	137 [117–162]	0.345
DBP, mmHg	81 [66–94]	78 [65–93]	76 [63–90]	0.143
Heart rate, bpm	87 [72–107]	90 [76–107]	86 [70–100]	0.042
Pre-hospitalization Katz index, score	6.00 [6–6]	6 [6–6]	6 [2–6]	<0.001
Hypertension (%)	58 (56.3)	136 (59.4)	114 (53.0)	0.407
Diabetes (%)	31 (30.1)	62 (27.7)	40 (18.6)	0.030
Dyslipidemia (%)	16 (23.9)	35 (23.3)	15 (11.7)	0.027
Atrial fibrillation (%)	36 (35.0)	100 (44.6)	92 (42.8)	0.247
Chronic kidney disease (%)	12 (17.9)	38 (25.3)	31 (24.2)	0.477
COPD (%)	6 (5.8)	14 (6.2)	11 (5.1)	0.876
Cerebrovascular disease (%)	13 (12.6)	39 (17.4)	52 (24.2)	0.034
Angina pectoris (%)	8 (11.9)	10 (6.7)	7 (5.5)	0.238
Myocardial infarction (%)	13 (12.6)	52 (23.2)	40 (18.6)	0.074
Heart failure admission (%)	36 (37.1)	90 (41.9)	84 (40.2)	0.731
Lower limb peripheral arterial disease (%)	1 (1.5)	8 (5.3)	5 (3.9)	0.413
Malignant tumors (%)	10 (14.9)	31 (20.7)	29 (22.7)	0.439
Care levels of Japanese public long-term care insurance (%)				<0.001
Nothing	95 (92.2)	150 (67.0)	78 (36.8)	
Support Level 1	4 (3.9)	21 (9.4)	30 (14.2)	
Support Level 2	3 (2.9)	16 (7.1)	13 (6.1)	
Care Levels 1	1 (1.0)	11 (4.9)	29 (13.7)	
Care Levels 2	0 (0.0)	17 (7.6)	23 (10.8)	
Care Levels 3	0 (0.0)	7 (3.1)	21 (9.9)	
Care Levels 4	0 (0.0)	2 (0.9)	13 (6.1)	
Care Levels 5	0 (0.0)	0 (0.0)	5 (2.4)	
Nursing home resident (%)	1 (1.0)	8 (3.6)	22 (10.2)	0.001
Employment status (%)	41 (39.8)	43 (19.2)	13 (6.0)	<0.001
Length of hospital stay, days	12 [9–17]	14 [11–19]	17 [13–25]	<0.001
Outcomes (%)				<0.001
Home or the original facility	98 (95.1)	208 (92.9)	171 (79.6)	
Transfer to another hospital	4 3.9)	6 (2.7)	19 (8.8)	
Admission to a facility	0 (0.0)	4 (1.8)	7 (3.3)	
In-hospital death	1 (1.0)	2 (0.9)	16 (7.4)	
Transfer to another department	0 (0.0)	4 (1.8)	2 (0.9)	
Grip strength (women), kg	18.2 [16.4–20.7]	15.2 [12.4–18.8]	12.3 [9.0–14.9]	<0.001
Grip strength (men), kg	31.4 [25.9–37.3]	26.1 [22.4–30.8]	21.0 [17.1–25.9]	<0.001
SPPB, score	10 [8–12]	7 [4–10]	3 [1–6]	<0.001

Data are presented as median [25%-75%] or number (%).

Alb: Albumin, BMI: Body mass index, BNP: Brain natriuretic peptide, COPD: Chronic obstructive pulmonary disease, CRP: C-reactive protein, DBP: Diastolic blood pressure, eGFR: Estimated glomerular filtration rate, GNRI: Geriatric Nutritional Risk Index, Hb: Hemoglobin, LVEF: Left ventricle ejection fraction, MCI: Mild cognitive impairment,, SBP: Systolic blood pressure, SPPB: Short physical performance battery.

The results of the cognitive function assessment at the time of discharge classified 90 patients (35.9%) into the maintenance group, 117 (46.6%) into the improvement group, and 44 (17.5%) into the decline group (Table 2). There was a statistically significant difference among the three groups only in cognitive function at admission (P = 0.026). Other patient characteristics were not significantly different (Table 2).

**Table 2:** Patient characteristics stratified by change in cognitive function.

	Maintenance group (N = 90)	Improvement group (N = 117)	Decline group (N = 44)	P value
Cause of hospitalization (%)				0.404
Heart failure	77 (85.6)	96 (82.1)	41 (93.2)	
Coronary artery disease	11 (12.2)	19 (16.2)	2 (4.5)	
Other	2 (2.2)	2 (1.7)	1 (2.3)	
Age, years	87 [80–91]	85 [77–89]	86 [82–90]	0.095
Women (%)	56 (62.2)	55 (47.0)	25 (56.8)	0.087
BMI, kg/m^2^	22.1 [18.6–24.0]	22.1 [19.4–25.2]	20.3 [18.8–23.5]	0.210
LVEF, %	48.5 [37.3–63.6]	52.0 [35.3–63.7]	54.3 [43.7–64.4]	0.761
Alb, g/dL	3.5 [3.3–3.8]	3.4 [3.1–3.7]	3.5 [3.1–3.8]	0.222
Hb, g/dL	11.4 [10.1–12.7]	11.5 [10.0–13.3]	11.4 [10.0–12.7]	0.657
BNP, pg/mL	465.6 [244.5–1063.5]	479.4 [210.7–924.5]	569.7 [351.1–1131.6]	0.298
CRP, mg/dL	0.8 [0.1–2.6]	1.1 [0.3–3.9]	0.8 [0.2–1.9]	0.161
eGFR, mL/min/1.73 m^2^	34.5 [24.6–49.3]	41.4 [27.8–54.6]	34.1 [25.4–46.6]	0.105
GNRI, score	93.8 [86.0–100.7]	92.4 [85.4–100.6]	90.2 [86.7–94.9]	0.462
SBP, mmHg	140 [116–165]	137 [115–160]	132 [108–158]	0.465
DBP, mmHg	81 [61–97]	77 [63–91]	72 [66–90]	0.495
Heart rate, bpm	85 [75–100]	87 [74–102]	80 [69–98]	0.507
Pre-hospitalization Katz index, score	6 [5–6]	6.00 [6–6]	6 [3–6]	0.224
Hypertension (%)	55 (61.1)	59 (50.4)	19 (43.2)	0.111
Diabetes (%)	22 (24.4)	30 (25.6)	6 (13.6)	0.255
Dyslipidemia (%)	14 (23.7)	16 (20.5)	5 (19.2)	0.861
Atrial fibrillation (%)	35 (38.9)	51 (43.6)	21 (47.7)	0.599
Chronic kidney disease (%)	18 (30.5)	18 (23.1)	7 (26.9)	0.619
COPD (%)	5 (5.6)	7 (6.0)	2 (4.5)	0.939
Cerebrovascular disease (%)	21 (23.3)	20 (17.1)	9 (20.5)	0.535
Angina pectoris (%)	1 (1.7)	5 (6.4)	1 (3.8)	0.400
Myocardial infarction (%)	18 (20.0)	25 (21.4)	6 (13.6)	0.539
Heart failure admission (%)	38 (43.2)	51 (44.3)	18 (40.9)	0.926
Lower limb peripheral arterial disease (%)	4 (6.8)	5 (6.4)	3 (11.5)	0.671
Malignant tumors (%)	14 (23.7)	19 (24.4)	3 (11.5)	0.366
Care levels of Japanese public long-term care insurance (%)				0.406
Nothing	40 (44.9)	61 (52.6)	23 (52.3)	
Support Level 1	12 (13.5)	17 (14.7)	4 (9.1)	
Support Level 2	7 (7.9)	6 (5.2)	2 (4.5)	
Care Levels 1	7 (7.9)	7 (6.0)	8 (18.2)	
Care Levels 2	11 (12.4)	11 (9.5)	1 (2.3)	
Care Levels 3	8 (9.0)	10 (8.6)	2 (4.5)	
Care Levels 4	3 (3.4)	2 (1.7)	3 (6.8)	
Care Levels 5	1 (1.1)	2 (1.7)	1 (2.3)	
Nursing home resident (%)	7 (7.8)	7 (6.0)	2 (4.5)	0.751
Employment status (%)	8 (8.9)	17 (14.5)	4 (9.1)	0.387
Length of hospital stay, days	18 [14–29]	19 [15–25]	20 [14–29]	0.703
Outcomes (%)				0.569
Home or the original facility	80 (88.9)	105 (89.7)	38 (86.4)	
Transfer to another hospital	6 (6.7)	6 (5.1)	5 (11.4)	
Admission to a facility	4 (4.4)	4 (3.4)	0 (0.0)	
In-hospital death	0 (0.0)	1 (0.9)	0 (0.0)	
Transfer to another department	0 (0.0)	1 (0.9)	1 (2.3)	
Grip strength (women), kg	12.8 [10.3–17.1]	12.9 [11.4–16.0]	12.3 [10.2–14.3]	0.415
Grip strength (men), kg	25.2 [22.1–27.8]	22.4 [19.0–27.3]	23.0 [18.5–27.4]	0.188
SPPB, score	6 [3–9]	5 [2–8]	4 [2–7]	0.329
Cognitive function (%)				0.026
Non-cognitive impairment	15 (16.7)	6 (5.1)	7 (15.9)	
MCI	37 (41.1)	45 (38.5)	12 (27.3)	
Cognitive impairment	38 (42.2)	66 (56.4)	25 (56.8)	

Data are presented as median [25%-75%] or number (%).

Alb: Albumin, BMI: Body mass index, BNP: Brain natriuretic peptide, COPD: Chronic obstructive pulmonary disease, CRP: C-reactive protein, DBP: Diastolic blood pressure, eGFR: Estimated glomerular filtration rate, GNRI: Geriatric Nutritional Risk Index, Hb: Hemoglobin, LVEF: Left ventricle ejection fraction, MCI: Mild cognitive impairment, SBP: Systolic blood pressure, SPPB: Short physical performance battery.

The results of the multivariate analysis of factors associated with changes in cognitive function during hospitalization are shown in Table 3. Multivariate analysis identified MCI at admission, cognitive decline at admission, and age as factors associated with cognitive improvement when the reference was cognitive maintenance (MCI, odds ratio (OR) = 5.62, 95% confidence interval (CI): 1.69–18.7; Cognitive impairment, OR = 12.3, 95% CI: 3.47–43.5; Age, OR = 0.96, 95% CI = 0.92–0.99). Sex, log-BNP, and length of hospital stay were not significantly associated with improved cognitive function when the reference was cognitive maintenance. With reference to maintenance of cognitive function, age, sex, log-BNP, days in hospital, and cognitive function at admission were not significantly associated with cognitive decline. Similarly, models were created to investigate laboratory data, comorbidities, pre-admission activity status, and physical function. However, only age and cognitive function at admission were associated with improved cognitive function during hospitalization, and no factors related to cognitive decline during hospitalization were extracted.

**Table 3:** Examination of factors associated with changes in cognitive function.

Reference: maintenance group	Improvement group			Decline group			Improvement group			Decline group			Improvement group			Decline group			Improvement group			Decline group		
	OR (95% CI)		P value		OR (95% CI)	P value	OR (95% CI)		P value		OR (95% CI)	P value	OR (95% CI)		P value		OR (95% CI)	P value	OR (95% CI)		P value		OR (95% CI)	P value
Non-cognitive impairment	reference			reference																				
MCI	5.62	(1.69–18.7)	<0.001	0.63	(0.19–2.09)	0.454	9.60	(2.55–36.1)	<0.001	0.82	(0.24–2.80)	0.747	5.62	(1.64–19.2)	0.006	0.56	(0.17–1.87)	0.349	6.13	(1.82–20.6)	0.003	0.61	(0.18–2.07)	0.424
Cognitive impairment	12.3	(3.47–43.5)	<0.001	1.38	(0.42–4.57)	0.595	16.6	(4.12–66.9)	<0.001	1.36	(0.39–4.75)	0.629	11.3	(3.10–41.4)	<0.001	1.16	(0.35–3.87)	0.813	9.48	(2.56–35.2)	<0.001	1.07	(0.29–3.98)	0.921
Age, years	0.96	(0.92–0.99)	0.013	1.01	(0.97–1.06)	0.659	0.95	(0.92–0.98)	0.004	1.01	(0.97–1.06)	0.533	0.94	(0.91–0.97)	<0.001	1.00	(0.96–1.05)	0.895	0.95	(0.91–0.99)	0.011	1.00	(0.95–1.06)	0.929
Man, vs women	1.89	(0.98–3.65)	0.058	1.57	(0.69–3.57)	0.285																		
Log-BNP	0.92	(0.70–1.22)	0.562	1.26	(0.87–1.83)	0.226																		
Length of hospital stay, day	0.99	(0.97–1.01)	0.391	1.00	(0.98–1.02)	0.993																		
Alb, g/dL							0.51	(0.26–1.00)	0.051	0.67	(0.29–1.58)	0.364												
Hb, g/dL							1.08	(0.93–1.24)	0.305	1.03	(0.86–1.23)	0.746												
eGFR, mL/min/1.73 m^2^							1.02	(1.00–1.04)	0.055	1.01	(0.99–1.03)	0.244												
Atrial fibrillation (%)													1.13	(0.60–2.15)	0.698	1.53	(0.69–3.40)	0.299						
Heart failure admission (%)													1.24	(0.65–2.34)	0.516	0.79	(0.35–1.77)	0.567						
Cerebrovascular disease (%)													0.52	(0.25–1.10)	0.088	0.85	(0.34–2.12)	0.727						
Employment status (%)																			1.43	(0.46–4.44)	0.538	1.41	(0.30–6.57)	0.661
Pre-hospitalization Katz index, score																			1.12	(0.93–1.36)	0.234	0.96	(0.77–1.21)	0.748
SPPB, score																			0.92	(0.83–1.03)	0.146	0.94	(0.82–1.09)	0.422

Alb: Albumin, BNP: Brain natriuretic peptide, CI: Confidence interval, eGFR: Estimated glomerular filtration rate, Hb: Hemoglobin, MCI: Mild cognitive impairment, OR: Odds ratio, SPPB: Short physical performance battery.

The background factors of individuals who dropped out of cognitive function assessment follow-up during hospitalization, compared to those of individuals who continued, are presented in Table 4. The dropout group had a higher proportion of younger individuals, males, those not requiring care, and those with higher BMI, higher Alb, higher Hb, lower BNP, lower CRP, higher eGFR, higher GNRI, higher pre-hospitalization Katz index, lower rate of lower limb peripheral arterial disease, higher employment rate, shorter length of hospital stay, higher in-hospital death rate, higher grip strength, higher SPPB score, and better cognitive function at admission.

**Table 4:** Characteristics of patients who dropped out and those who continued follow-up.

	Dropout (N = 291)	Follow-up (N = 251)	P value
Cause of hospitalization (%)			
Heart failure	228 (78.4)	214 (85.3)	0.051
Coronary artery disease	59 (20.3)	32 (12.7)	
Other	4 (1.4)	5 (2.0)	
Age, years	82 [72–88]	85 [80–90]	<0.001
Women (%)	128 (44.0)	136 (54.2)	0.020
BMI, kg/m^2^	23.0 [20.4–25.9]	21.9 [19.1–24.7]	0.001
LVEF, %	54.1 [36.7–64.9]	52.1 [37.0–64.0]	0.637
Alb, g/dL	3.6 [3.3–3.9]	3.5 [3.2–3.8]	0.001
Hb, g/dL	11.9 [10.3–13.8]	11.4 [10.0–12.9]	0.008
BNP, pg/mL	417.0 [180.2–692.8]	490.5 [244.4–1057.0]	<0.001
CRP, mg/dL	0.5 [0.1–2.6]	0.9 [0.2–3.0]	0.018
eGFR, mL/min/1.73 m^2^	44.7 [29.5–58.0]	38.9 [25.4–50.6]	0.002
GNRI, score	96.9 [89.8–105.6]	92.2 [85.6–99.4]	<0.001
SBP, mmHg	137 [118–161]	137 [113–160]	0.745
DBP, mmHg	78 [65–93]	77 [63–93]	0.569
Heart rate, bpm	88 [72–107]	86 [74–101]	0.150
Pre-hospitalization Katz index, score	6 [6–6]	6 [5–6]	0.002
Hypertension (%)	172 (59.1)	133 (53.0)	0.165
Diabetes (%)	75 (25.8)	58 (23.1)	0.485
Dyslipidemia (%)	31 (17.0)	35 (21.5)	0.338
Atrial fibrillation (%)	121 (41.6)	107 (42.6)	0.862
Chronic kidney disease (%)	38 (20.9)	43 (26.4)	0.253
COPD (%)	17 (5.8)	14 (5.6)	1.000
Cerebrovascular disease (%)	54 (18.6)	50 (19.9)	0.743
Angina pectoris (%)	18 (9.9)	7 (4.3)	0.060
Myocardial infarction (%)	56 (19.2)	49 (19.5)	1.000
Heart failure admission (%)	103 (37.6)	107 (43.3)	0.211
Lower limb peripheral arterial disease (%)	2 (1.1)	12 (7.4)	0.005
Malignant tumors (%)	34 (18.7)	36 (22.1)	0.503
Care levels of Japanese public long-term care insurance (%)			
Nothing	199 (68.6)	124 (49.8)	<0.001
Support Level 1	22 (7.6)	33 (13.3)	
Support Level 2	17 (5.9)	15 (6.0)	
Care Levels 1	19 (6.6)	22 (8.8)	
Care Levels 2	17 (5.9)	23 (9.2)	
Care Levels 3	8 (2.8)	20 (8.0)	
Care Levels 4	7 (2.4)	8 (3.2)	
Care Levels 5	1 (0.3)	4 (1.6)	
Nursing home resident (%)	15 (5.2)	16 (6.4)	0.581
Employment status (%)	68 (23.4)	29 (11.6)	<0.001
Length of hospital stay, days	12 [9–16]	19 [14–27]	<0.001
Outcomes (%)			
Home or the original facility	254 (87.3)	223 (88.8)	<0.001
Transfer to another hospital	12 (4.1)	17 (6.8)	
Admission to a facility	3 (1.0)	8 (3.2)	
In-hospital death	18 (6.2)	1 (0.4)	
Transfer to another department	4 (1.4)	2 (0.8)	
Grip strength (women), kg	15.3 [12.0–18.3]	12.8 [10.7–16.1]	0.001
Grip strength (men), kg	29.0 [23.3–34.5]	23.8 [19.2–27.7]	<0.001
SPPB, score	8 [4–11]	5 [2–8]	<0.001
Cognitive function (%)			<0.001
Non-cognitive impairment	75 (25.8)	28 (11.2)	
MCI	130 (44.7)	94 (37.5)	
Cognitive impairment	86 (29.6)	129 (51.4)	

Data are presented as median [25%–75%] or number (%).

Alb: Albumin, BMI: Body mass index, BNP: Brain natriuretic peptide, COPD: Chronic obstructive pulmonary disease, CRP: C-reactive protein, DBP: Diastolic blood pressure, eGFR: Estimated glomerular filtration rate, GNRI: Geriatric Nutritional Risk Index, Hb: Hemoglobin, LVEF: Left ventricle ejection fraction, MCI: Mild cognitive impairment, SBP: Systolic blood pressure, SPPB: Short physical performance battery.

## Discussion

Cognitive impairment in patients with cardiovascular diseases is associated with poor prognosis, increased risk of rehospitalization, and functional outcomes. Prevention of cognitive impairment is crucial for disease management. However, the actual changes in cognitive function during hospitalization in patients with cardiac disease and the factors associated with these changes are unknown. To the best of our knowledge, tThis study is the first to elucidate the changes in cognitive function during hospitalization for patients with cardiovascular disease and to investigate their characteristics. The novelty of this research lies in the two-point assessment during hospitalization, which revealed that 46.6% of patients with cardiovascular disease experienced cognitive improvement, whereas 17.5% had cognitive decline. Those with MCI or cognitive impairment at admission, as well as younger patients, were associated with improved cognitive function during hospitalization.

Prior longitudinal studies assessing MMSE in outpatient populations indicated a decline of -0.9 points over one year [28] and -0.2 points over 1.5 years in community-dwelling older adults [29]. Cooley et al. reported a -0.9-point decline in MoCA scores over one year in a longitudinal study [30]. Studies conducted in Japan targeting older adults reported that 30% experienced a decrease of ≥2 points in MoCA-J scores, whereas 30% showed an improvement over one year [22]. In contrast, in the present study, those with cognitive decline at admission were likelier to have improved cognitive function at discharge.

Cerebral blood flow improves with treatment in patients with cardiovascular disease [11,12], and studies on healthy individuals have indicated improved cognitive function with increased cerebral blood flow [13–15]. Based on prior studies on community-dwelling individuals, it can be inferred that patients with cardiovascular disease exhibit improved cognitive function more efficiently. This may be due to correcting a temporary decrease in cerebral blood flow caused by cardiac disease through treatment, resulting in improved cognitive function. However, this study did not evaluate cerebral blood flow and other factors, which presents a future research opportunity.

In our study, 17.5% of patients experienced a decline in cognitive function during hospitalization. Older adults in the hospital have a high prevalence of delirium and depressive symptoms [9], with a 2.4 times greater likelihood of cognitive decline [31]. Older patients with cardiovascular disease often have lower ADL capabilities at admission and lower activity levels in the ward [32], making them more susceptible to the effects of bed rest during hospitalization. Furthermore, patients with cardiovascular disease may experience reduced physical activity due to intravenous treatment [33]. The association between physical activity and cognitive function has been revealed in numerous studies [34,35], and it is plausible that the decline in cognitive function observed in our study was influenced by bed rest during hospitalization. This study has a small sample size and limited variables due to its retrospective nature. Therefore, future prospective studies are needed to examine in detail the factors related to changes in cognitive function.

Considering the results of this study, a certain number of patients with cardiovascular disease will experience cognitive decline during hospitalization, making screening tests for cognitive function important. Interventions that maintain or improve cognitive function should then be considered. Targeted interventions for these individuals could contribute to improved cognitive function, prevention of rehospitalization, and better life prognosis. However, it is essential to note that older patients with cardiovascular disease require individualized focus, and guidelines emphasize the importance of providing comprehensive cardiac rehabilitation interventions tailored to each patient [6]. Individualized assessments, especially risk assessments for cognitive function changes during hospitalization, and interventions by specialized teams are crucial.

The strength of this study is that it reveals previously unknown changes in cognitive function during hospitalization, which is essential for long-term prognosis and disease management in patients with cardiac disease. However, this study has several limitations, including its small sample size and single-center setting. Future large-scale multicenter cohort studies are necessary to elucidate the factors related to cognitive function changes during hospitalization in patients with cardiovascular diseases. We performed a multivariate analysis, but overfitting problems limited the number of independent variables. Future studies should use large data sets and conduct detailed analyses using multivariate and subgroup analyses. In such studies, investigating additional factors related to cognitive function, such as physical activity, quality of life, education level, delirium, and depressive symptoms, is essential. Furthermore, the high dropout rate in our study suggests the possibility of sampling bias. The patients analyzed in this study may represent a more severe subset of patients with cardiovascular disease, as those who dropped out had fewer complications and were discharged earlier. Efforts to evaluate the factors contributing to dropout and establish measures to minimize dropout are needed. Therefore, high-quality prospective studies are required in the future. Additionally, the data in our study are limited to the hospitalization period, and the post-discharge course is unknown. Because changes in cognitive function during hospitalization could be temporary, further detailed investigations are required to determine whether cognitive decline during hospitalization is associated with cognitive decline, the onset of dementia or MCI after discharge, as well as with life prognosis, rehospitalization, and functional prognosis. Finally, this study did not consider the effects of treatment during hospitalization. Future studies should include a more detailed examination of treatment and the resulting changes in cardiac function.

## Conclusions

On admission, 41.3% of patients with cardiovascular disease exhibited cognitive impairment, and 40.2% had MCI. Cognitive decline occurred in 17.5% of the patients during hospitalization, whereas cognitive improvement occurred in 46.6%. These findings demonstrate the importance of assessing cognitive changes in hospitalized patients with cardiovascular disease. Future studies are needed to identify factors associated with changes in cognitive function.
